# Editorial: From Physiological Adaptations to Endurance Performance: It Is Time to Bridge the Gap

**DOI:** 10.3389/fspor.2021.775654

**Published:** 2021-10-12

**Authors:** Franck Brocherie, Kazushige Goto, Olivier Dupuy, Mathieu Gruet, Fabrice Vercruyssen, Julien Louis

**Affiliations:** ^1^Laboratory Sport, Expertise and Performance (EA 7370), French Institute of Sport (INSEP), Paris, France; ^2^Faculty of Sport and Health Science, Ritsumeikan University, Shiga, Japan; ^3^University of Poitiers, Laboratory MOVE (EA 6314), Faculty of Sport Sciences, Poitiers, France; ^4^School of Kinesiology and Physical Activity Sciences, University of Montreal, Montreal, QC, Canada; ^5^IAPS Laboratory, University of Toulon, Toulon, France; ^6^Research Institute for Sport and Exercise Sciences, Liverpool John Moores University, Liverpool, United Kingdom

**Keywords:** exercise physiology, cycling, running, sport performance, performance testing, endurance

## Endurance Performance: A Vast Worksite

At first glance, endurance performance appears relatively simple as it relates to the efficiency of the aerobic metabolism through the cascade of oxygen transfer from inspiration of atmospheric oxygen down to tissue mitochondria (Hoppeler, 2018). Yet, despite the profusion of research on endurance performance (i.e., >30,000 “endurance physiology”-related studies available on Pubmed within a century), there is a mismatch between endurance performance *per se* and its underlying physiological mechanisms. Indeed, studies rarely link athletes' performance/profile and their physiological adaptation in response to a given training or ergogenic intervention. In order to identify and understand the physiological adaptations that directly translate into enhanced endurance performance, this Research Topic intended to mobilize the scientific community to connect physiological adaptations [e.g., maximal oxygen consumption (${\dot{\text{V}}\text{O}}_{2\text{max}}$)], mitochondrial content) with endurance performance (e.g., power output, time, distance) in a single- or multi-disciplinary sports (e.g., marathon or triathlon).

## The Research Topic Articles

Developed through three sections (i.e., elite sports and performance enhancement, exercise physiology, sport and exercise nutrition) and three Frontiers journals (i.e., Frontiers in Sports and Active Living, Frontiers in Physiology, Frontiers in Nutrition) that reflect the editors' aspiration for an integrative/multidisciplinary approach, this Research Topic includes eight peer-reviewed articles (Aandahl et al., De Larochelambert et al., Durand and Raberin, Gronwald et al., Lember et al., Malgoyre et al., Sumi et al., van der Zwaard et al.) From the themes initially listed in the call for papers, these resulting articles address endurance performance in the light of physiological/molecular adaptation, altitude training, energy/glycogen availability and nutritional strategies/periodization. Population and sports covered include Olympic/elite athletes, well- and recreationally-trained males and females performing either rowing, running or triathlon, with the exception of a single study which involved female Wistar rats.

Building on the conceptual framework proposed by Joyner and Coyle (2008), van der Zwaard et al. proposed a meta-analysis that highlights the interrelationships between endurance performance and its key macroscopic (i.e., ${\dot{\text{V}}\text{O}}_{2\text{max}}$, lactate threshold and efficiency/economy) and microscopic (e.g., skeletal muscle fiber type and size, capillarization) physiological determinants. The authors also highlight several potential training-induced adaptations to maximize endurance performance and encourage the integrative assessment of physiological determinants during endurance-based training sessions. For instance, Gronwald et al. reported the usefulness of real-time alpha1 of Detrended Fluctuation Analysis—a non-linear heart-rate variability index based on fractal correlation properties that may be used as a proxy of homeodynamic regulation and network physiology during endurance exercise (Gronwald and Hoos, 2020)—for aerobic threshold estimation and exercise intensity distribution in a former Olympic triathlete.

Besides physiological properties, other aspects (e.g., biomechanics, psychology) likely impact endurance performance. As such, De Larochelambert et al. weighted the anthropometric indicators related to pacing performance for each Olympic rowing category and sex. Given that anthropometric indicators are linked to ${\dot{\text{V}}\text{O}}_{2\text{max}}$, peak power, power resistance, buffering agent, muscle typology, muscle coordination, and biomechanical determinants (e.g., Izquierdo-Gabarren et al., 2010), differentiating the anthropometrical impacts in particular parts of races may be helpful to better understand rowing performance. In running disciplines, performance also arises from a combination of optimal anthropometrical, physiological and behavioral characteristics (Lieberman and Bramble, 2007). Considering the limited data on the physiological and metabolic implications of participating in the increasingly popular hill running discipline, Lember et al. characterized the associations between anthropometric variables, aerobic capacity, running performance, energy intake and expenditure in hill runners from different ages. Their results indicate that aerobic capacity positively impacts hill running performance, while adiposity and age negatively affects it. Of importance, hill runners appear at increased risk of negative energy availability (Mountjoy et al., 2018) which may predispose to physiological dysfunction or adaptive alteration.

Among the ergogenic aids available to enhance athletes' endurance performance, carbohydrate (CHO) availability before, during and after training is now accepted as a potential regulator of cell signaling pathways, training adaptations and exercise performance (Impey et al., 2018). Aandahl et al. demonstrated that a high-CHO pre-event meal (3 g·kg^−1^) led to higher peak velocity and longer time to exhaustion compared to exercising in a fasted state or after a low-CHO pre-event meal (0.5 g·kg^−1^), both in well- and recreationally-trained rowers, runners and triathletes. Although CHO oxidation was higher (as inferred through higher rate of exchange ratio) in both groups following the high-CHO pre-event meal, the main whole-body physiological determinants of endurance performance were not impacted. Hence, CHO intake should be standardized when testing exercise capacity, whereas it seems of lesser importance when whole-body physiological determinants are assessed.

Since the 1968 Olympic Games, altitude training (see the updated panorama of the different hypoxic methods in Girard et al., 2017) has been increasingly used by athletes to enhance physiological adaptation and athletic performance. Malgoyre et al. used an animal model to investigate the effects of 5 weeks “living high-training high” paradigm (simulated altitude of 3,200 m) on aerobic performance and quantitative and qualitative mitochondrial changes in the *plantaris* muscle. Their findings indicate that mitochondrial function and substrate utilization do not contribute to the higher “living high-training high”-induced aerobic performance in long-distance running. This highlights the need to appropriately manage the “hypoxic dose” to promote expected hypoxia-induced physiological responses (e.g., increase in hemoglobin mass) while preventing any impediment of mitochondrial adaptation. In this view, Sumi et al. reported that three consecutive days of relatively matched endurance training performed either under hypoxia or normoxia similarly improved endurance capacity, but with lower subjective feeling of fatigue and without increased risk of overtraining syndrome or injury when using hypoxia. Finally, Durand and Raberin discussed the consequences of the exercise-induced hypoxemia phenomenon (Dempsey et al., 1984) in endurance athletes in the particular context of altitude/hypoxic training. The current knowledge on the interaction between the physiological determinants of exercise-induced hypoxemia and the hypoxic dose requires more exploration to draw practical recommendations.

## Concluding Remarks

The array of contents presented in this Research Topic is illustrative of the endless interest in endurance performance. We also believe that calls for new research in the area (not limited to the themes we proposed) should be renewed to better appreciate the contemporary challenges that face endurance athletes. In this line, there is no doubt that continuous advances in technologies and the increasing sports sciences support to athletes will facilitate this initiative and make physiological measurements in ecological settings even more accurate and insightful.

## Author's Note

We hope that the readers will enjoy, and benefit from reading this collection. Feedback are most welcome.

## Author Contributions

All authors listed have made a substantial, direct and intellectual contribution to the work, and approved it for publication.

## Conflict of Interest

The authors declare that the research was conducted in the absence of any commercial or financial relationships that could be construed as a potential conflict of interest.

## Publisher's Note

All claims expressed in this article are solely those of the authors and do not necessarily represent those of their affiliated organizations, or those of the publisher, the editors and the reviewers. Any product that may be evaluated in this article, or claim that may be made by its manufacturer, is not guaranteed or endorsed by the publisher.
